# Genome-Wide Characterization of Alternative Splicing Events and Their Responses to Cold Stress in Tilapia

**DOI:** 10.3389/fgene.2020.00244

**Published:** 2020-03-18

**Authors:** Bi Jun Li, Zong Xian Zhu, Hui Qin, Zi Ning Meng, Hao Ran Lin, Jun Hong Xia

**Affiliations:** State Key Laboratory of Biocontrol, Institute of Aquatic Economic Animals and Guangdong Provincial Key Laboratory for Aquatic Economic Animals, College of Life Sciences, Sun Yat-sen University, Guangzhou, China

**Keywords:** alternative splicing, tilapia, cold stress, RNA-seq, genome

## Abstract

Alternative splicing (AS) is an important post-transcriptional regulatory mechanism for cells to generate transcript variability and proteome diversity. No systematic investigation of AS events among different tissues in response to stressors is available for tilapia currently. In this study, AS among different tissues was identified and the cold stress-related AS events were explored in a Nile tilapia (*Oreochromis niloticus*) line based on 42 RNA-seq datasets using a bioinformatics pipeline. 14,796 (82.76%; SD = 2,840) of the expression genes showed AS events. The two most abundant AS types were alternative transcription start site (TSS) and terminal site (TTS) in tilapia. Testis, brain and kidney possess the most abundant AS gene number, while the blood, muscle and liver possess the least number in each tissue. Furthermore, 208 differentially alternative splicing (DAS) genes in heart and 483 DAS in brain in response to cold stress. The number of AS types for alternative exon end, exon skipping and retention of single intron increased significantly under cold stress. GO enrichment and pathway overrepresentation analysis indicated that many DAS genes, e.g., genes in circadian clock pathway, may influence expression of downstream genes under cold stress. Our study revealed that AS exists extensively in tilapia and plays an important role in cold adaption.

## Introduction

Alternative splicing (AS) generates multiple functional mRNAs and therefore enables different proteins to be synthesized from one single gene (Maniatis and Tasic, 2002; Nilsen and Graveley, 2010). This regulatory mechanism significantly expands transcript variability and proteome diversity, as well as provides additional information in eukaryotic cells (Nilsen and Graveley, 2010). AS events widely exist in eukaryotes (Sorek, 2007). For example, 92–94% of human genes undergo regulation of AS (Wang et al., 2008). Many AS events are well conserved and appear to be related to the phylogenetic distance among species (Mastrangelo et al., 2012; Jonsson et al., 2016). AS participates in organogenesis (Anand et al., 2008), development (Yamaguchi and Iwasa, 2018), tissue identity (Baralle and Giudice, 2017; Cong et al., 2018), disease occurrence (Scotti and Swanson, 2016; Martinez-Montiel et al., 2018) and heavy metals, cold and heat, pathogen attacks (Christensen et al., 1992; Hopf et al., 1992; Ali and Reddy, 2008; Mazzucotelli et al., 2008) in eukaryotes.

The flexibility of AS contributes to environmental adaption and phenotypic plasticity in organism (Mastrangelo et al., 2012). When environmental fluctuations are ubiquitous, mRNA can be processed into new isoforms with shifting expression level, so that cells can alter their metabolism and maintain homeostasis (Kucherenko and Shcherbata, 2018). For example, in duck, an AS event in *mitf* gene causes down-regulation of melanogenesis pathway, which led to plumage color change (Zhou Z. et al., 2018). The different isoforms from peptidoglycan recognition proteins (PGRPs) are required in recognition of different bacteria (Chang and Zhang, 2017). Stress-related AS events have been identified in many species, such as mild thermal, oxidative and heavy metal stress in yeast (Melangathli et al., 2017), temperature, salinity and air exposure stress in Pacific oyster (Huang et al., 2016; Liu and Guo, 2017), chemical stress or thermal stress in *Drosophila* (Fujikake et al., 2005; Kim et al., 2014; Ruan et al., 2015; Jaksic and Schlotterer, 2016), IFN antiviral response in zebrafish (Zhang et al., 2018), heat shock in pigs (Ju et al., 2014) and stress treatment in human cells (Jacob et al., 2014), *Edwardsiella ictaluri* infection in catfish (Tan et al., 2018) and resistance to iridovirus in Asian seabass (Wang et al., 2017).

The high resolution of RNA-seq data allows for exploring not only gene expression but also genome-wide AS events in animals. RNA-seq has been extensively used for measuring gene expression to gain biological insights in aquatic animals recently (Chatchaiphan et al., 2017; Im et al., 2017; Gan et al., 2018; Shi et al., 2018; Wang et al., 2018; Yang et al., 2018; Ye et al., 2018; Yu et al., 2018; Yue et al., 2018). In teleost, some studies highlighted the important role of AS in some genes (Marchand et al., 2001; Chan and Cheng, 2004; Moore et al., 2005). AS frequencies ranging from 17% of mapped genes in zebrafish to ∼43% of mapped genes in the pufferfish were estimated using the public EST/cDNA datasets (Lu et al., 2010). However, only few studies paid attention to the AS responses to stress in fish (Zwollo, 2011; Fillatreau et al., 2013; Long et al., 2013; Wan and Su, 2015; Chang and Zhang, 2017; Tan et al., 2019). Further deep mining of transcriptomic data using bioinformatic tools to identify AS events will help clarify gene regulation mechanism under stressors in fish.

Tilapia is one of the most important agricultural fish worldwide (El-Sayed, 2006). Currently, Nile tilapia (*Oreochromis niloticus*), blue tilapia (*Oreochromis aureus*), Mozambique tilapia (*Oreochromis mossambicus*) and various hybrids including red tilapia are the major cultured species/strains worldwide (Xia et al., 2015; Chen et al., 2019). The gene expression changes in response to different stressors including cold stress (Hu et al., 2016), alkalinity stress (Zhao et al., 2015), salinity adaptation (Xu et al., 2015), hypoxia stress (Li et al., 2017b; Wang et al., 2017; Xia et al., 2018), cortisol stress (Tsalafouta et al., 2018), thermal stress (Ventura et al., 2018; Yue et al., 2018), oxidative stress (Giannetto et al., 2017), and immune response (Li et al., 2017d) have been explored by RNA-seq in tilapia. Genome-wide identification of copy number variation (Li et al., 2017a), QTL intervals for hypoxia and salt tolerance traits (Li et al., 2017c; Gu et al., 2018) and long non-coding RNAs (Yue et al., 2018) have been performed. In tilapia, AS events were explored for hypoxia stress based on RNA-seq datasets (Melangathli et al., 2017; Xia et al., 2018) and nutrition status based on gene cloning (He et al., 2014) recently. No systematic investigation of AS events among different tissues in response to stressors is available for tilapia.

Cold is a severe stressor to fish in aquatic environment. Most of cold-related AS studies are focused on plants (James et al., 2012; Pose et al., 2013), insects (Majercak et al., 1999) and mammals (Gemignani et al., 2002; Sano et al., 2015). In zebrafish, differential expression analysis found that RNA splicing is one of the key biological processes; and spliceosome is a highly enriched pathway for genes that up-regulated under cold stress (Long et al., 2013). Moreover, AS and promoter switching of genes are found to be regulated under cold stress (Long et al., 2013). Several studies have paid attention to characterize the transcriptional responses to cold stress in fish brain or heart. For example, differential gene expression was explored in the brain of the channel catfish (*Ictalurus punctatus*) (Ju et al., 2002). Previous studies also identified temperature acclimation in the heart transcriptome of the rainbow trout (*Oncorhynchus mykiss*) (Vornanen et al., 2005). There studies indicate the importance of these tissues in the cold stress response in fish.

Although the importance of AS mechanisms in animal stress response has been extensively reported, surprisingly few studies (Xu et al., 2015) have surveyed AS events in fish at genome-wide level. Here, we characterized genome-wide AS events among ten different tissues and then identified cold stress-related AS events in brain and heart of a Nile tilapia line based on 42 RNA-seq datasets using a bioinformatics pipeline. Our findings suggested widely existence of AS events in tilapia and their important roles in cold adaptation.

## Materials and Methods

### Fish Management, Cold Treatment, and Sample Collection

Thirty-six Nile tilapia individuals (GIFT line; ∼90 dph) were randomly selected from a population raised at the fish facility of School of Life Sciences, Sun Yat-sen University. These fishes were evenly divided into two indoor conical fiber glass tanks (water depth: 70 cm, volume: 500 L) in a recirculating freshwater system for acclimation of 2 days with water temperature at 25–28°C and dissolved oxygen (DO) > 6 mg L^–1^. The fishes were fed with tilapia pellets twice a day and the photoperiod was adjusted to 12 D:12 L in the room.

During low temperature treatment, the fishes in one tank were used as the control and those in the other were considered as test samples. The temperature in the water of test tank was slowly decreased to 15°C within 12 h using a water cooler. To investigate the AS changes under cold stress, six Nile tilapia in test tank were then cultured in 15°C for 24 h, other six in control tank were maintained at 26°C. The detailed information on stress treatment was stated in Li et al. (Yue et al., 2018). During treatment, no feed was given to fish. Tricaine mesylate (MS-222) is used for euthanasia of fish. A dose of ∼ 400 ppm for immersion of the fish for ∼10 min is usually applied. Brain and heart tissues were collected from each fish of each tank. The tissue samples were frozen immediately in liquid nitrogen and then stored at −80°C until RNA isolation.

### RNA Isolation and NGS Sequencing

Total RNA from cold-treated samples was isolated using TRIzol reagent (Invitrogen, United Kingdom) according to the manufacturer’s protocol. The RNA quality was assessed using the Nanodrop-2000 (Thermo Fisher Scientific, United States) and electrophoresis in 1.5% agarose gel. Total RNA integrity was further evaluated using Bioanalyzer 2100 (Agilent Technologies). Every two samples from the same tissue of the same group were pooled equally. Twelve libraries from heart and brain samples that constructed by TruSeq^TM^RNA sample preparation kit according to the product instruction (Illumina) were finally sequenced using Illumina HiSeq2500 for pair-end (PE) 150 sequencing.

### Identification of AS Events From RNA-seq Datasets

To identify AS events in different tissues of Nile tilapia, we downloaded 30 Nile tilapia RNA-seq data from the NCBI database^1^. The 30 RNA-seq datasets (Supplementary Additional File 1) were generated from 10 tissues (testis, brain, kidney, eye, ovary, skin, heart, blood, muscle, and liver). For each tissue, three replicates were available for AS identification.

Both RNA-seq datasets including the 12 samples that generated in our experiment and the 30 samples downloaded from NCBI database were aligned to the tilapia reference genome (Orenile1.0; downloaded from the Ensembl database)^2^ using the software TopHat (Trapnell et al., 2009). The read alignments of RNA-seq data were conducted using SAMtools (Li et al., 2009). Aligned reads were then assembled using Cufflinks (Trapnell et al., 2012) and compared to the tilapia reference annotation using Cuffcompare (Trapnell et al., 2010). The “extract-as” program of ASprofile software package was applied to determine AS events among multiple samples (Florea et al., 2013) using the *gtf* file that generated by cufflinks and the *tmap* file that generated by cuffcompare. The result of ASprofile was sorted by 12 AS types including SKIP, IR, AE, transcription start site (TSS), alternative transcription terminal site (TTS), MSKIP, XSKIP, XMSKIP, XIR, MIR, XMIR, and alternative exon ends (XAE). Significance test of AS types among 10 tissues was performed using *t* statistics in Microsoft Office Excel.

### AS Events in Response to Cold Stress

TopHat software (Trapnell et al., 2009) can identify novel transcripts from locations of regions and estimate abundance of the transcripts from their depth of coverage in the mapping. Bam files that generated by TopHat were used to identify differential AS (DAS) in response to cold stress. We processed the aligned RNA-seq data using QoRTs package (Hartley and Mullikin, 2015). Once alignment and quality control were completed for the dataset, the coverage counts (for genes, exons, and known/novel splice-junctions) were generated via QoRTs (Hartley and Mullikin, 2015). DAS events between groups were then analyzed using JunctionSeq (Hartley and Mullikin, 2016). False discovery rate (FDR) value of 0.01 was used as the significant threshold for each comparison. Gene structure was displayed using online tool Gene Structure Display Server 2.0 (Hu et al., 2015) and using the software FasParser (Sun, 2017), and protein structure of AS genes was plotted with online tool SMART (Letunic et al., 2015).

### Different Expression Gene Analysis

Different expression gene analysis of brain and heart samples were conducted using the cuffdiff program (Trapnell et al., 2013). The generated DEG data were then filtered using the parameters: | log2(fold_change)| > 1 and the summary count values for each gene in two datasets >20 and FDR ≤ 0.05.

### GO Annotation and Pathway Enrichment

The Ensembl gene IDs for both DAS and DEG genesets were transferred to Gene symbols in David website^3^. We carried out overrepresentation test (Released 20181113) for both DAS and DEG datasets by using the online tool Panther database^4^ (PANTHER version 14.0 released on 2018-12-03) and the zebrafish geneset as the reference list. Several analysis types were applied for the datasets including PANTHER GO-Slim biological process, molecular function, cellular component, PANTHER protein class and Reactome pathways. The Fisher’s exact test was performed with FDR correction and a FDR of <0.05.

We also investigated the enrichment of the affiliated genes in the GO Molecular Functions, GO Biological Processes and GO Cellular Components datasets, using the online software Metascape^5^ (Tripathi et al., 2015). The genes were annotated using the default resources that provided by Metascape. The *p* value cutoff was set to 0.01.

### RT-PCR Analysis

RT-PCR analysis was performed to verify AS events. Primer pairs (Supplementary Additional File 2) for 10 selected exon-skipped events that predicted by Junctionseq were designed using NCBI primer designing tool^6^. The primers were located in the upper and lower exons of the targeted one. RNA was treated with DNase I to avoid the contamination effect of genomic DNA. Reverse transcription was carried out using the reverse transcription kit (TAKARA PrimeScript^TM^RT reagent Kit with gDNA Eraser) according to the manufacturer’s instructions. PCR amplifications were conducted in a 20 μL volume using a 2 × PCR mastermix (Dongsheng, China) and 10 μg cDNA, 1 μL of forward and reverse primers (10 μM) in a Bio-Rad cycler. The following program was applied including one cycle of 3 min at 94°C, 35 cycles of 30 s at 94°C, 30 s at 60°C and 30 s at 72°C, followed by a final extension of 10 min at 72°C. The PCR products were detected by running 2.5% agarose electrophoresis. All reactions were conducted in triplicate.

## Results

### Genome-Wide Identification of AS Events in Different Tissues of Nile Tilapia

To obtain an overview of AS events in tilapia genome, 30 RNA-seq datasets were downloaded from the NCBI database (Supplementary Additional File 1). These datasets consisted of RNA-seq reads from 10 tissues, including testis, brain, kidney, eye, ovary, skin, heart, blood, muscle, and liver. For each tissue, three replicates were available. The total base number of reads in our data sets was 158.8 Gb and the average mapping ratio was 78.9% (70.9–88.0%).

**FIGURE 1:**
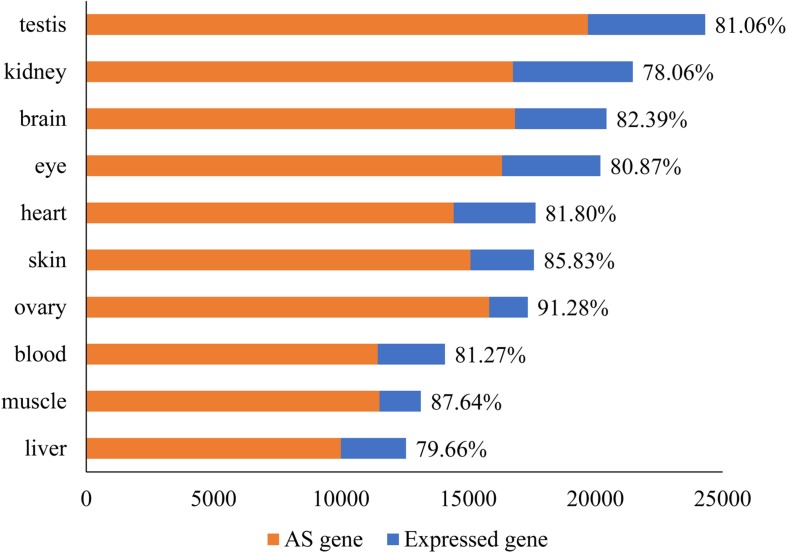
The number of expressed genes and DAS in every tissue of tilapia. The number of expressed genes in 10 tissues ranged from 12,559 to 24,209. The number of genes under AS ranged from 10,005 to 19,705. The percentage of DAS in all expressed genes was given after the bar.

Only successfully mapped high quality reads to the reference genome were used for *ab initio* transcriptome assembly with cufflinks. From the assembly, cuffcompare and ASprofile were applied to predict AS events in different samples. The average number of expressed genes in 10 tissues was 17,878 (SD = 3,628). Surprisingly, 14,796 (SD = 2,840) of the genes (82.76%) showed AS events (Figure 1). Ovary (91.28%) and muscle (87.64%) had the highest rate of AS, while kidney (78.06%) and liver (79.66%) had the lowest rate, compared to the number of expressed genes in each tissue.

**FIGURE 2:**
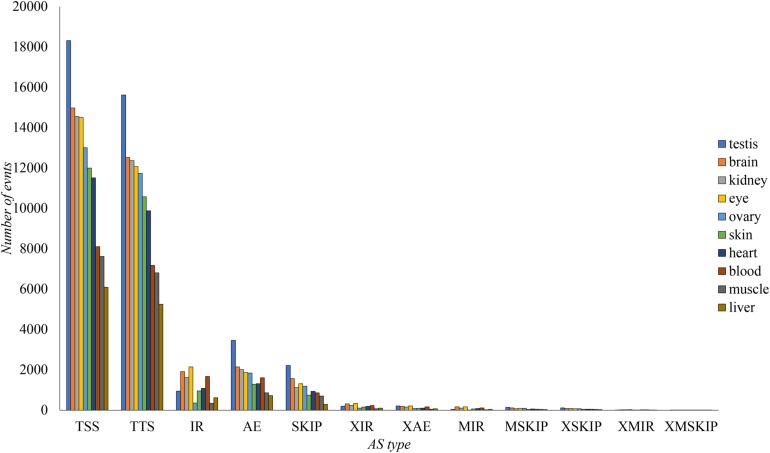
Classification of different AS events in every tissue of tilapia. The AS number in ten tissues for each type was shown. Exon skipping (SKIP), retention of single intron (IR), alternative exon end (AE), alternative transcription start site (TSS), alternative transcription terminal site (TTS), continuous exons skipping (MSKIP), multiple discontinuous exons skipping (XSKIP), multiple continuous exons skipping (XMSKIP), corresponding introns retention events (XIR, MIR, XMIR), and alternative exon ends (XAE).

The AS events in transcriptome were classified into 12 types according to their structures (Florea et al., 2013). The numbers of AS events for 10 tissues were calculated based on the 30 NCBI RNA-seq datasets (Figure 2). Testis had the most abundant AS events (51,285), while the number in liver was the lowest (23,246).

TSS and TTS were the most abundant AS types in all tissues (Figure 2). These events were likely to be located in untranslated region (UTR), and might have smaller influence to final protein sequences than other types, e.g., retention of single intron (IR), exon skipping (SKIP), and alternative exon end (AE). However, the 5′ UTR and 3′ UTR were also essential in biology process, involved into the modulation of messenger RNA (mRNA) transcription, secondary structure, stability, localization, translation, and accessed to regulators like microRNAs (miRNAs) and RNA-binding proteins (RBPs) (Leppek et al., 2018; Steri et al., 2018).

The tissue-specific AS events were rare. One tissue specific DAS gene was found for heart, kidney, liver, and ovary respectively; and seven specific AS genes were identified in skin (Supplementary Additional File 3). Most of gene splicing patterns were shared by at least two tissues. However, much more work needs to be done to validate the results of bioinformatics analysis.

### AS Events Identified in Tilapia Under Cold Stress

To investigate the AS regulation in response to cold, 12 RNA-seq data was generated for a Nile tilapia line under cold stress. These data were available at the DDBJ database (BioProject Accession: PRJDB6721). In brain, a total of 483 DAS genes were found in response to cold stress, including 426 exons and 527 junctions with significant differentially expressed counts (*p* < 0.01). In heart, the DAS number was 208, with 166 differentially expressed exons and 178 differentially expressed junctions (*p* < 0.01) (Supplementary Additional File 4).

**FIGURE 3:**
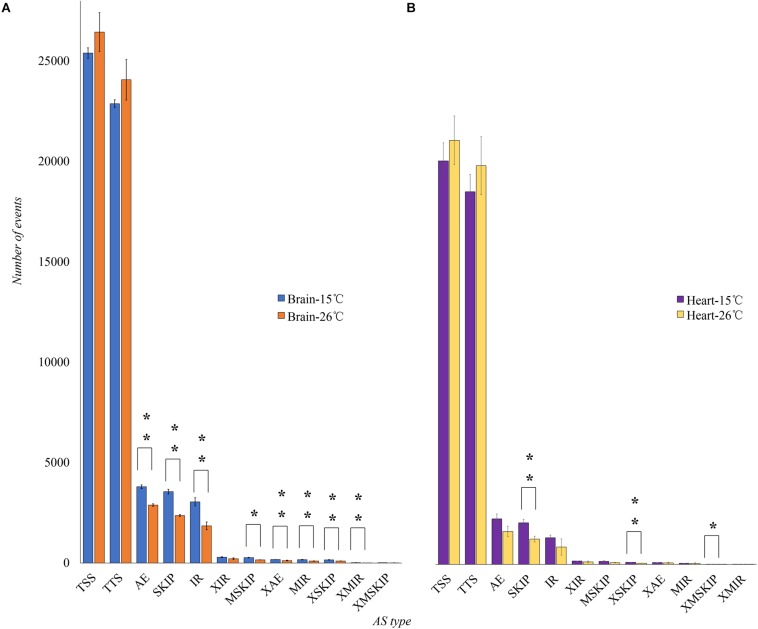
The number of AS events in brain and heart of tilapia in response to cold stress. We compared the number of AS genes between the control group (26°C) and the test group (16°C). **(A)** ^∗^Showed the number of AS events increases significantly in brain (*p* < 0.01). **(B)** ^∗^Showed the AS events increase significantly in heart (*p* < 0.01).

In brain, the number of AE, SKIP, IR, continuous exons skipping (MSKIP), alternative exon ends (XAE), multiple discontinuous exons skipping (XSKIP), and corresponding introns retention events (MIR, XMIR) of AS events significantly increased in response to cold stress (*p* < 0.01) (Figure 3A). In heart, the SKIP, XSKIP and XMSKIP events increased significantly in response to cold stress (*p* < 0.01) (Figure 3B). These events were likely to cause amino acid changes in proteins.

Reactome pathway enrichment analysis found that DAS genes in mRNA Splicing – Major Pathway (FDR = 2.73E-02, fold enrichment = 4.54), mRNA splicing pathway (FDR = 3.07E-02, fold enrichment = 4.33) and Processing of Capped Intron-Containing Pre-mRNA (FDR = 5.37E-03, fold enrichment = 4.42) were overrepresented in brain. However, in heart, DAS genes were overrepresented in much more pathways, e.g., metabolism of RNA (FDR = 1.76E-04, fold enrichment = 4.54), translation (FDR = 2.16E-05, fold enrichment = 8.17), eukaryotic translation initiation (FDR = 1.76E-04, fold enrichment = 4.54), major pathway of rRNA processing in the nucleolus and cytosol (FDR = 1.26E-05, fold enrichment = 10.17) (Table 1).

**TABLE 1:** Reactome pathway overrepresentation analysis of DAS events in brain and heart of Nile tilapia under cold stress.

**Tissue value**	**Reactome pathways**	**Gene number**	**Fold enrichment**	**FDR**
brain	mRNA Splicing – major pathway	11	4.54	2.73E-02
	Processing of capped intron-containing pre-mRNA	14	4.42	5.37E-03
	mRNA splicing	11	4.33	3.07E-02
heart	RHO GTPases activate PAKs	3	24.41	3.49E-02
	SRP-dependent cotranslational protein targeting to membrane	10	20.1	3.38E-07
	Non-sense mediated decay (NMD) independent of the exon junction complex (EJC)	10	19.2	2.56E-07
	Formation of a pool of free 40S subunits	10	17.62	3.70E-07
	Non-sense mediated decay (NMD) enhanced by the exon junction complex (EJC)	10	16.27	5.65E-07
	Non-sense-mediated decay (NMD)	10	16.27	4.52E-07
	GTP hydrolysis and joining of the 60S ribosomal subunit	10	15.12	7.29E-07
	L13a-mediated translational silencing of ceruloplasmin expression	10	15.12	6.25E-07
	Cap-dependent translation initiation	10	14.12	1.01E-06
	Eukaryotic translation initiation	10	14.12	8.98E-07
	Major pathway of rRNA processing in the nucleolus and cytosol	10	10.17	1.51E-05
	rRNA processing	10	10.17	1.37E-05
	rRNA processing in the nucleus and cytosol	10	10.17	1.26E-05
	Translation	11	8.17	2.16E-05
	Metabolism of RNA	15	4.54	1.76E-04

*Pathways with FDR < 0.05 were showed in the table. Gene number, fold enrichment and FDR values were given.*

Panther protein classification found that the DAS genes in brain were enriched in multiple categories including mRNA processing factor (FDR = 3.93E-04, fold enrichment = 5.95), RNA binding protein (FDR = 3.06E-02, fold enrichment = 2.4) and mRNA processing factor (FDR = 3.93E-04, fold enrichment = 5.95), while the DAS genes in heart were enriched in the category of ribosomal protein (FDR = 2.91E-02, fold enrichment = 7.02), RNA binding protein (FDR = 6.24E-05, fold enrichment = 4.55), and actin family cytoskeletal protein (FDR = 3.66E-02, fold enrichment = 4.48) (Supplementary Additional File 5).

**FIGURE 4:**
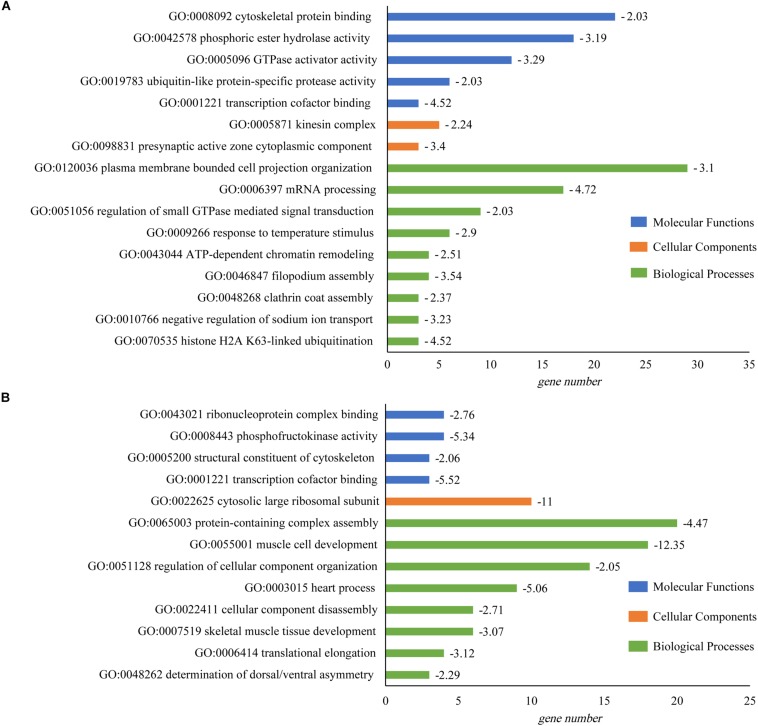
GO enrichment of DAS identified in tilapia heart and brain. **(A)** GO enrichment of DAS genes in brain. **(B)** GO enrichment of DAS genes in heart. The horizontal axis showed the gene number in every pathway; and value of log_10_(P) was given after the bar.

GO enrichment analysis suggested that many GO categories e.g., mRNA processing (GO:0006397) were significantly enriched in brain. The plasma membrane bounded cell projection organization (GO:0120036) in the biological process category had the most abundant DAS genes. In heart, DAS genes were significantly enriched in multiple categories including protein-containing complex assembly (GO:0065003), muscle cell development (GO:0055001) and heart process (GO:0003015). Protein-containing complex assembly (GO:0065003) had the largest number of DAS genes (20, Log_10_(P) = −4.47). Gene number and fold enrichment of every GO term were shown in the Figure 4.

### RT-PCR Validation of DAS Genes

To validate the accuracy and reliability of analysis process, RT-PCR was applied to measure expression level of different transcripts from one gene. For exon skipping or intron retention events, the primer pairs were designed in the upper exon and lower exon of the events, so that nucleotide fragments with different length could be amplified by PCR if there exists AS (Supplementary Additional File 2).

**FIGURE 5:**
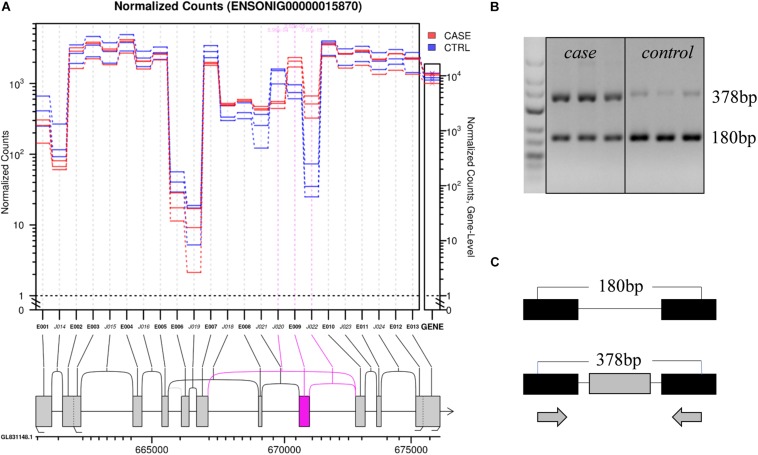
An example showing structure of the gene ENSONIG00000018570 and RT-PCR validation of its expression. **(A)** Visualization of gene structure. The ninth exon of gene ENSONIG00000018570 showed higher expression level in the case group (cold-treated). **(B)** Agarose electrophoresis of RT-PCR products. The 378 bp band was more abundant in the case group indicating the higher level of exon-included isoform under cold. **(C)** The location of primers on the gene. The gray bars represented the exons which underwent differential splicing (targeted) and the black bars represented the exons near the targeted one. The arrows indicated the primer location.

For example, in gene ENSONIG00000015870, an exon skipping event occurred in response to cold stress. The exon-included PCR product was 378 bp, while the exon-excluded DNA fragment was 180 bp. As seen from Figure 5, the exon-including transcripts were more abundant in cold-treated group (case) than the control group (control). The result was consistent with bioinformatics analysis.

**FIGURE 6:**
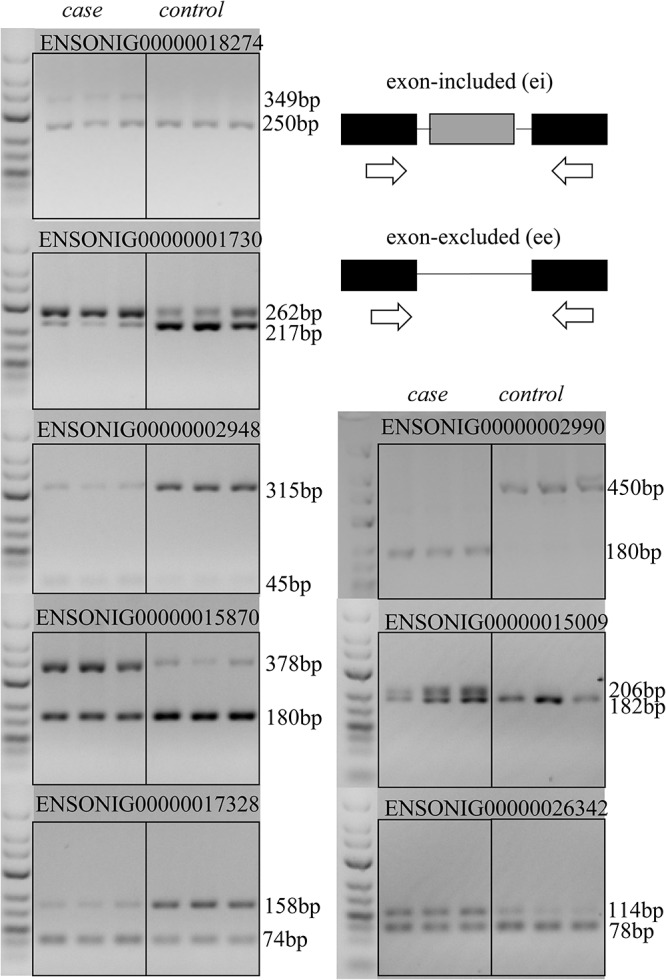
RT-PCR validation of AS events that predicted in RNA-seq data. RT-PCR validation was performed for AS events in eight genes that predicted by bioinformatic analysis. Except for the gene ENSONIG00000002990 producing a ∼450 bp band which was longer than the predicted size 211 bp, the results for other seven genes were as expected.

The RT-PCR result of 8 DAS genes in brain and heart revealed that most of AS events (7/8) that predicted in our experiment was credible, except for the gene ENSONIG00000002990 in which an unexpected 450 bp sequence was amplified, but a 211 bp fragment was supposed (Figure 6). This may be originated from individual specific AS events.

### Different Expression Genes Under Cold Stress

Alternative splicing events affected expression of downstream genes (Zhou Z. et al., 2018). To investigate the effect to downstream genes, we further carried out a DEG analysis of our transcriptome datasets. There are 2,918 DEGs in brain and 1,673 DEGs in heart in response to cold stress (15°C) (Supplementary Additional File 6).

In brain, significant overrepresentation of DEGs was found in 13 Reactome pathways, e.g., metabolism (FDR = 1.62E-07, fold enrichment = 1.58), mRNA splicing (FDR = 1.21E-02, fold enrichment = 2.27), cell cycle (FDR = 2.82E-02, fold enrichment = 1.67), metabolism of lipids (FDR = 3.30E-04, fold enrichment = 1.81), and metabolism of protein (FDR = 9.47E-03, fold enrichment = 1.39) (Supplementary Additional File 7). There were 11 protein class categories were significantly overrepresented including RNA binding protein, transaminase, isomerase, oxidoreductase (Supplementary Additional File 8).

In heart, DEGs were enriched in 33 Reactome pathways, e.g., metabolism (FDR = 5.83E-12, fold enrichment = 1.97), signal transduction (FDR = 5.31E-03, fold enrichment = 1.45), transport of small molecules (FDR = 1.63E-04, fold enrichment = 2.06), gene expression (transcription) (FDR = 4.93E-02, fold enrichment = 1.61), and cell cycle (FDR = 4.81E-02, fold enrichment = 1.82) pathway in the reactome pathway category (Supplementary Additional File 6). Seven protein classes were enriched, mainly including transaminase, metalloprotease, oxidoreductase, and RNA binding protein (Supplementary Additional File 7).

**FIGURE 7:**
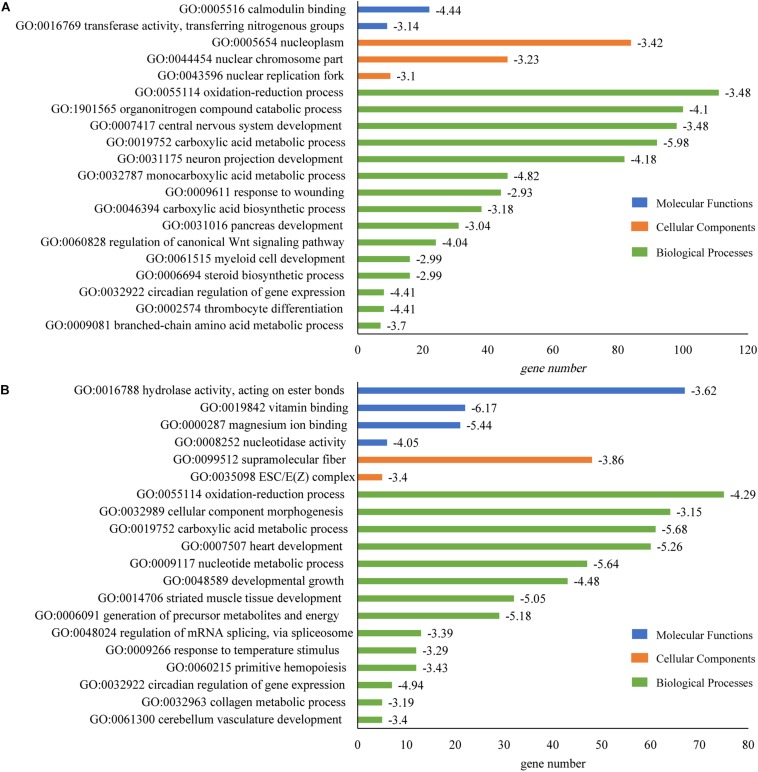
GO enrichment of DEGs that identified in tilapia heart and brain. **(A)** GO enrichment of DEGs in brain. **(B)** GO enrichment of DEGs in heart. The horizontal axis showed the gene number in every pathway; and value of log_10_(P) was given after the bar.

It was observed that DEGs were significantly enriched in 15 categories of biological processes, including carboxylic acid metabolic process (GO:0019752; log_10_(P) = −5.98), monocarboxylic acid metabolic process (GO:0032787; log_10_(P) = −4.82), thrombocyte differentiation (GO:0002574; log_10_(P) = −4.41), circadian regulation of gene expression (GO:0032922; log_10_(P) = −4.41) and neuron projection development (GO:0031175; log_10_(P) = −4.18) in brain (Figure 7A).

In heart, DEGs were significantly enriched in 14 categories of biological processes. The most enriched pathways were carboxylic acid metabolic process (GO:0019752; log_10_(P) = −5.68), nucleotide metabolic process (GO:0009117; log_10_(P) = −5.64), heart development (GO:0007507; log_10_(P) = −5.26), generation of precursor metabolites and energy (GO:0006091; log_10_(P) = −5.18) and striated muscle tissue development (GO:0014706; log_10_(P) = −5.05) (Figure 7B). The most DEG genes were found in the oxidation-reduction process (GO:0055114) for both brain (111 genes) and heart tissues (76 genes) (Figures 7A,B).

### Links Between DAS and DEG in Response to Cold Stress

**FIGURE 8:**
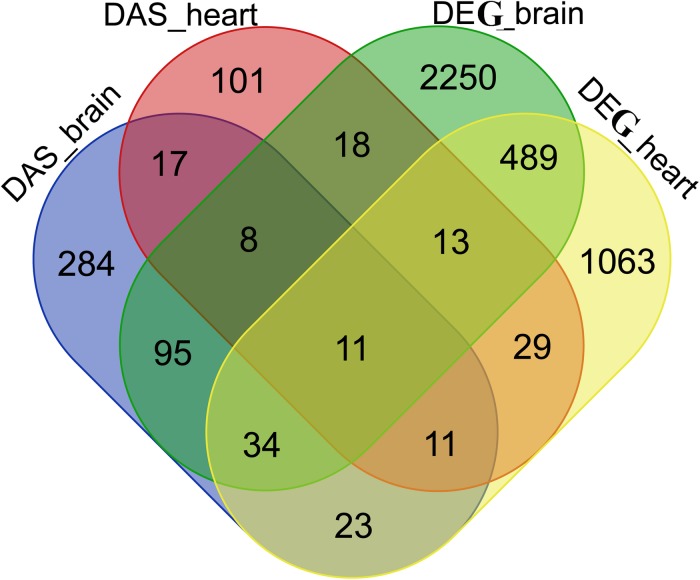
Venn diagram of DASs and DEGs in tilapia brain and heart. There are 208 DAS genes in heart, 483 DAS genes in brain, 2,918 DEGs in brain and 1,673 DEGs in heart.

It was observed that many gene overlaps between DAS and DEG datasets. For example, 148 genes were shared between brain DAS and DEG datasets and 64 genes shared between heart DAS and DEG datasets. A total of 11 genes were shared among 4 datasets, including ENSONIG00000005637, ENSONIG00000008480 ENSONIG 00000004328, ENSONIG00000017643, ENSONIG00000018614, ENSONIG00000019320, ENSONIG00000015833, ENSONIG0 0000014686 ENSONIG00000011498, ENSONIG00000011813, and ENSONIG0000000410. The information on overlapping between DAS and DEG datasets were presented in Figure 8.

**FIGURE 9:**
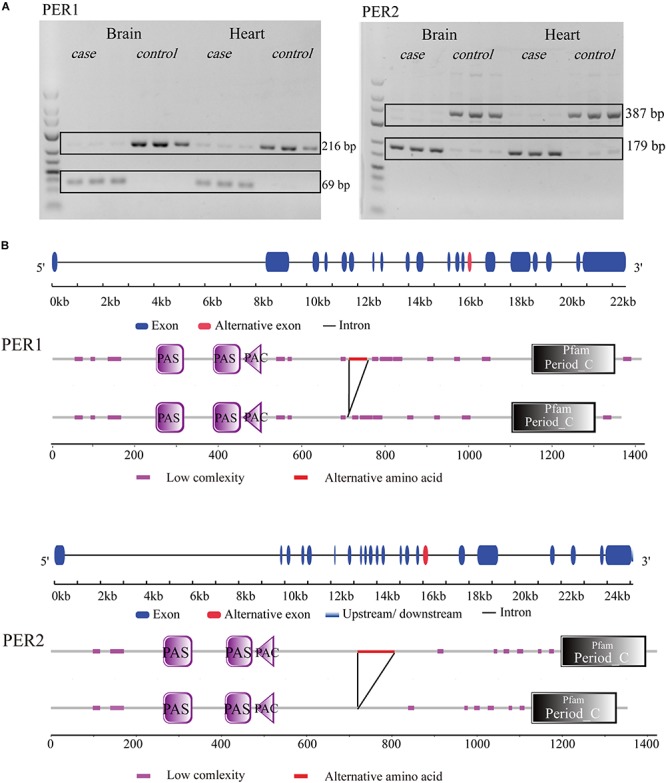
Gene and protein structure of *per* gene in tilapia. **(A)** *per1* and *per2* genes had exon skipping in brain and heart under cold stress. **(B)** In cold, *per1* lose one 147 bp exon fragment (in red) and *per2* lose one 208 bp exon fragment in CDS (in red). Both AS events caused changes of amino acid sequences in proteins (in red).

There were many DAS and DEG genes, which could be classified into the same pathway categories (Supplementary Additional File 9). Interestingly, there was a pathway, circadian regulation of gene expression (GO:0032922), with enrichment of both DAS and DEG genes in heart and brain in response to cold stress. Three DAS genes (*per3*, *per2*, *per1a*) and eight DEG genes in brain (*clocka*, *per3*, *per2*, *bhlhe40*, *nfil3-5*, *nr1d2a*, *nr1d1*, *per1a*) and heart (*clocka*, *per3*, *per2*, *bhlhe40*, *nfil3-5*, *nfil3-6*, *nr1d1*, *nampta*) were classified into this category. The gene list was shown in Supplementary Additional File 8. Among these, *per1* and *per2* genes showed exon skipping both in brain and heart under cold stress. An 147bp exon in per1 was cut off (Figure 9A). For *per2*, a 208bp exon was spliced, which leaded to a deletion in CDS (Figure 9B). Both AS events caused a deficiency of a part of amino acid sequences in proteins (Figures 9A,B).

## Discussion

### Developing a Reference Dataset for Studying on AS Function in Tilapia

Alternative splicing was of great importance in improving regulatory capacities and proteomic complexities in eukaryotes (Ben-Dov et al., 2008). In teleost, some studies highlighted the important role of AS in a few genes, e.g., thyroid hormone receptors in teleost fish (Marchand et al., 2001; Chan and Cheng, 2004; Moore et al., 2005). However, few studies carried out genome-wide characterization of AS events in fish (Lu et al., 2010; Long et al., 2013). In this study, we explored the genome-wide AS events in 10 tissues of Nile tilapia based on 42 RNA-seq datasets. This represented the largest datasets used in the characterization of AS events in tilapia.

In Nile tilapia, the average percentage of AS genes was 82.76% with a range from 78.06% (kidney) to 91.28% (ovary) across 10 tissues. The value was comparable to that in mammal species. For example, 94.4–95.5% of expressed genes in ovary of pig (Tang et al., 2018) and about 70% of genes in human might be alternatively spliced (Johnson et al., 2003). Surprisingly, the value of AS frequency in tilapia was far more than that identified in reported fish species. The AS frequency from 17% of mapped genes in zebrafish to ∼43% of mapped genes in the pufferfish were identified based on a collective EST and CDS datasets from public database (Lu et al., 2010). The low frequencies may be mainly caused from the smaller datasets used in two studies. Further analysis based on larger datasets from different tissues may identify more AS expectedly.

The high abundance of AS numbers in tilapia testis and brain was similar to human (Yeo et al., 2004; Elliott and Grellscheld, 2006). Relatively fewer AS events were found in tilapia liver when compared to the findings in other tissues. The reason behind this might be the well-differentiation and low-complexity of liver tissue (Yeo et al., 2004). We also found 208 DAS genes in heart and 483 DAS genes in brain in response to cold stress. Under cold stress, the number of AE, SKIP and IR increased significantly. Our first investigation of genome-wide AS patterns across 10 tissues of Nile tilapia based on a large dataset provides a basis for study on AS function in tilapia.

### mRNA Splicing Might Play an Important Part in Temperature Adaptation

Alternative splicing plays important roles in phenotype determination and biological response to environmental stress. The cold-related DEG analysis had been reported in liver and kidney (Yang et al., 2015; Zhou T. et al., 2018) in previous studies, and heart and brain of tilapia in this study. These studies indicated that cold stress might affect many pathways including metabolism and immunity in tilapia. However, cold-related RNA splicing was not deeply investigated in tilapia.

Splice site selection was under complex regulation by various cis-regulatory elements in pre-mRNAs and trans-regulatory elements. Many RBPs were shown to be critically required for the regulation of AS, such as serine/arginine-rich (SR) and heterogeneous nuclear RNP (hnrnp) protein families (Nilsen and Graveley, 2010; Wang et al., 2015). In the DAS gene list of catfish under heat stress, there were five SR genes (*srsf2*, *srsf3*, *srsf5*, *srsf7-like*, and *srsf11-like*) and two heterogeneous *hnrnp* genes (*hnrnpm* and *hnrnpa0*) (Tan et al., 2019). In zebrafish, RNA splicing was listed as one of the most highly overrepresented biological processes, while spliceosome was one of the most highly enriched pathways for genes up-regulated under cold stress (Long et al., 2013). These findings suggested that RBPs act in AS-related stress regulation in fish.

In our cold treatment to tilapia, pathway enrichment analysis found overrepresentation of DAS and DEG genes in RNA splicing pathway. In spliceosome pathway, many SR splicing factors were regulated under cold stress. For example, *srsf1, srsf3, srsf5* and *srpk3* underwent AS regulation. Besides, *srsf3*, *srsf5, srsf6, srpk2* genes showed differential expression level. In this pathway, *ddx5* gene was spliced differentially under cold stress in both brain and heart. Its complementary, *rbm8a*, was up-regulated in brain and heart. Several downstream genes were also influenced, such as *prpf* and *slu7* (Supplementary Additional File 2). Hnrnp protein families (*hnrnpc*, *hnrnpd*, *hnrnph3*, *hnrnpll*, and *hnrnpu*) also had differential expression level. Therefore, temperature stress affected the expression and splicing pattern of many genes in the spliceosome pathway of tilapia.

### Circadian Clock Pathway Might Play Important Roles in Cold Adaptation

Alternative splicing in *per* gene family was ubiquitous in many species. In zebrafish, alternative promoter usage was detected for *per3* gene under cold stress, which leads to a highly up-regulated transcript encoding a truncated protein lacking the C-terminal domains (Long et al., 2013). Different AS of *per* also existed in honeybees (Takeda et al., 2004).

*Per* gene was a key gene in the circadian clock pathway, whose protein product combined with CRY protein to suppress the expression of *arntl*/*clock* genes (Zambon et al., 2003). ARNTL:CLOCK protein complex went on to regulate the expression of *per* gene and *cry* gene. These composed a closed cycle pathway for biological rhythm. In the DEG datasets, the expression level of *arntl* and *clock* were both up-regulated. The change in per protein sequences might influence the function of arntl/clock protein, therefore weaked the suppression to gene expression of *arntl* and *clock*.

The relationship between circadian clock pathway and temperature was proved in previous study (Kidd et al., 2015). When *Drosophila* adapted to seasonally cold days, a thermo-sensitive splicing event in 3′ UTR played an important role (Majercak et al., 1999). In *Drosophila*, *per* generated two transcript types with an alternative intron in the 3′ UTR which was a key regulation on how the *Drosophila* circadian clock adapted to changes in temperature (Cheng et al., 1998; Majercak et al., 2004). The timeless (*tim*) gene in *Drosophila melanogaster* had two different transcript variants that were thermo-sensitively spliced (Boothroyd et al., 2007; Montelli et al., 2015). In zebrafish, alternative promoter usage was detected for *per3* gene after exposure to cold stress (16°C) for 24 h (Long et al., 2013).

The heme biosynthesis was believed to provide a key connection as a signaling molecule to indicate the nutritional status to the circadian oscillatory machinery and allow it to respond appropriately (Burris, 2008). In our DEG dataset, three genes (*alas1, cpox, fech*) in this pathway had decreased expression levels. According to our result, the rate-limiting enzyme of heme biosynthesis, *alas1*, had differential AS pattern and down-regulated expression level in heart. In organism, heme was a compound that played an important role in oxygen carrying. We speculated that the response of heme biosynthesis was related to oxygen consumption rate under cold.

Genes acting in heme were also regulated. *Rev-erb*α (also known as *NR1D1*) was a heme sensor that regulated the metabolic gene pathway, thus serving for coordination of circadian and metabolic pathways (Yin et al., 2010). *REV-ERB*α expression was regulated by the circadian genes binding to E-boxes within the Rev-erbα promoter. In our research, expression of *nr1d1* was significantly up-regulated in brain. *nr1d4b* and *nr1d2a* were also up-regulated in both brain and heart. Neuronal PAS domain protein 2 (NPAS2) was a heme-binding protein and played a role in the regulation of circadian rhythms. The DNA binding activity of NPAS2 was related to heme (Dioum et al., 2002). Heme could also combine with NR1D protein to repress expression of *arntl* and *npas2*. In our result, the expression of *npas2* was decreased in heart.

Rev-erbα response element was also responsive to *PPAR*α (Canaple et al., 2006). *PPAR*α played a pivotal role in the regulation of lipid homeostasis, fatty acid oxidation and acted as a key nutritional and environmental sensor for metabolic adaptation (Contreras et al., 2013; Seok and Cha, 2013). In our result, *PPAR*α had differential AS pattern under cold stress in heart. The role of *PPAR*α was a master regulator of lipid metabolism via regulation of numerous genes (Kersten, 2014). The down-regulation of most genes in cholesterol biosynthesis pathway, which indicated the close relationship between metabolic changes and fluctuations in biological rhythms.

## Conclusion

Alternative splicing was an essential mechanism in stress response. In this study, we characterized the genome-wide AS events in ten different tissues and identified cold tolerant related AS in heart and brain of Nile tilapia based on 42 RNA-seq datasets. Our findings suggest the widely existence of AS and its important roles in cold adaptation in tilapia. Further functional studies on AS events will help clarifying gene regulation mechanism under stress in tilapia.

## Data Availability Statement

All the read data under cold stress were available at the DDBJ database (BioProject Accession: PRJDB6721). All other datasets have been presented within the article/Supplementary Material.

## Ethics Statement

All experiments in this study were approved by the Animal Care and Use Committee of the School of Life Sciences at Sun Yat-sen University and were performed according to the regulations and guidelines established by this committee.

## Author Contributions

JX and HL contributed to the project conception. BL, ZZ, HQ, and ZM conducted the experiment and data analysis. JX and BL prepared the manuscript. All authors read and approved the final manuscript.

## Conflict of Interest

The authors declare that the research was conducted in the absence of any commercial or financial relationships that could be construed as a potential conflict of interest.

## Footnotes

1 https://www.ncbi.nlm.nih.gov/

2 www.ensembl.org

3 https://david.ncifcrf.gov/

4 www.pantherdb.org

5 http://metascape.org

6 www.ncbi.nlm.nih.gov/tools/primer-blast/index.cgi

## Abbreviations

AE: alternative exon end
AS: alternative splicing
DAS: differentially alternative splicing
DEGs: different expression genes
FDR: false discovery rate
hnrnp: heterogeneous nuclear RNP
IR: retention of single intron
miRNAs: microRNAs
mRNA: messenger RNA
MS-222: tricaine mesylate
MSKIP: continuous exons skipping
PGRPs: peptidoglycan recognition proteins
RBPs: RNA-binding proteins
SKIP: exon skipping
SR: serine/arginine-rich
TSS: transcription start site
TTS: alternative transcription terminal site
UTR: untranslated region
XAE: alternative exon ends
XIR, MIR XMIR: corresponding introns retention events
XMSKIP: multiple continuous exons skipping
XSKIP: multiple discontinuous exons skipping.
