# Supplementary material for: Genome-Wide Characterization of Alternative Splicing Events and Their Responses to Cold Stress in Tilapia
Source: Front Genet. 2020 Mar 18;11:244. doi: 10.3389/fgene.2020.00244 (PMC7093569; doi:10.3389/fgene.2020.00244)
Supplement: Supplementary file 2 [file Table_2.DOC]

**Additional file 2. The primer information for RT-PCR analysis**

| **Ensembl ID** | **Primer Sequence** | **Product Length (bp)** | |
| --- | --- | --- | --- |
|  |  | **Exon-excluded** | **Exon-included** |
| ENSONIG00000002990 | CCTTAGCAAGGGAACGGACG | 180 | 211 |
|  | TTTAGAAAACAAGGGGCTCGGT |  |  |
| ENSONIG00000015009 | GTGAGACGGTCACCACAGAG | 182 | 206 |
|  | CAGTAACTGGGCTGTTCCCA |  |  |
| ENSONIG00000026342 | GGTGGGACGGACATTCCTTT | 78 | 114 |
|  | CTATTCCCGATGCCGCAAAA |  |  |
| ENSONIG00000001730 | CTGATGGCCTTGCTGAACCT | 217 | 262 |
|  | AGGGCTTCCTGAGACTCACT |  |  |
| ENSONIG00000002948 | TGCCAGAGCCAGTCAAAAA | 45 | 315 |
|  | TTTCCATCAGCAGGGTCCTC |  |  |
| ENSONIG00000015870 | AGATCTCCACCAAGCCAACG | 180 | 378 |
|  | TCCTTGATGGGCAGAACACC |  |  |
| ENSONIG00000017328 | CCCCAGATCATCCACGGTTT | 74 | 158 |
|  | TGAACTCGAGGCATGCAGAG |  |  |
| ENSONIG00000018274 | TTAACAGGAGCATCCCGAGC | 250 | 349 |
|  | TACGGGAAAATCGAGGTGGC |  |  |
| ENSONIG00000009248 (Per1) | GACAAACATGAGCCCAACCG | 250 | 349 |
|  | CAGCGTGGGAGTTGTAGGAG |  |  |
| ENSONIG00000009815 (Per2) | ACAAACAGAAGGGGTCCAGC | 69 | 216 |
|  | CTGCCAAAACCTGCTTGGTC |  |  |
