# Supplementary material for: Genome-Wide Characterization of Alternative Splicing Events and Their Responses to Cold Stress in Tilapia
Source: Front Genet. 2020 Mar 18;11:244. doi: 10.3389/fgene.2020.00244 (PMC7093569; doi:10.3389/fgene.2020.00244)
Supplement: Supplementary file 5 [file Table_5.DOC]

| **Tissue** | **PANTHER Protein Class** | **Gene number** | **Fold enrichment** | **FDR** |
| --- | --- | --- | --- | --- |
| Brain | ribonucleoprotein | 5 | 8.49 | 3.29E-02 |
|  | RNA binding protein | 20 | 2.4 | 3.06E-02 |
|  | mRNA splicing factor | 8 | 5.51 | 1.74E-02 |
|  | mRNA processing factor | 12 | 5.95 | 3.93E-04 |
|  | C2H2 zinc finger transcription factor | 0 | < 0.01 | 4.53E-02 |
| Heart | ribosomal protein | 6 | 7.02 | 2.91E-02 |
|  | RNA binding protein | 17 | 4.55 | 6.24E-05 |
|  | actin family cytoskeletal protein | 8 | 4.48 | 3.66E-02 |

**Additional file 5. Overrepresentation of DAS in categories of PANTHER protein class**
