# Supplementary material for: Genome-Wide Characterization of Alternative Splicing Events and Their Responses to Cold Stress in Tilapia
Source: Front Genet. 2020 Mar 18;11:244. doi: 10.3389/fgene.2020.00244 (PMC7093569; doi:10.3389/fgene.2020.00244)
Supplement: Supplementary file 7 [file Table_7.DOC]

**Additional file 7.** **Reactome pathway overrepresentation analysis of DEGs detected in tilapia in response to cold stress**

| **Tissue** | **Reactome pathways** | **Gene Number** | **Fold Enrichment** | **FDR** |
| --- | --- | --- | --- | --- |
| Brain | Histidine, lysine, phenylalanine, tyrosine, proline and tryptophan catabolism | 13 | 3.43 | 4.30E-02 |
|  | Metabolism of amino acids and derivatives | 48 | 2.32 | 3.21E-04 |
|  | Metabolism | 223 | 1.58 | 1.62E-07 |
|  | mRNA Splicing - Major Pathway | 33 | 2.31 | 1.14E-02 |
|  | mRNA Splicing | 34 | 2.27 | 1.21E-02 |
|  | Processing of Capped Intron-Containing Pre-mRNA | 39 | 2.08 | 1.88E-02 |
|  | Metabolism of RNA | 70 | 1.6 | 3.93E-02 |
|  | S Phase | 27 | 2.28 | 3.07E-02 |
|  | Cell Cycle, Mitotic | 61 | 1.69 | 3.27E-02 |
|  | Cell Cycle | 67 | 1.67 | 2.82E-02 |
|  | Metabolism of lipids | 90 | 1.81 | 3.30E-04 |
|  | Post-translational protein modification | 135 | 1.42 | 2.18E-02 |
|  | Metabolism of proteins | 179 | 1.39 | 9.47E-03 |
|  | Unclassified | 1053 | 0.81 | 3.18E-26 |
| Heart | The role of GTSE1 in G2/M progression after G2 checkpoint | 10 | 3.97 | 3.44E-02 |
|  | G2/M Transition | 21 | 3.04 | 5.40E-03 |
|  | Mitotic G2-G2/M phases | 21 | 3 | 5.47E-03 |
|  | Cell Cycle, Mitotic | 37 | 1.82 | 4.81E-02 |
|  | Collagen biosynthesis and modifying enzymes | 13 | 3.89 | 1.37E-02 |
|  | Collagen formation | 14 | 2.9 | 4.48E-02 |
|  | Extracellular matrix organization | 29 | 2.11 | 2.75E-02 |
|  | Autodegradation of Cdh1 by Cdh1:APC/C | 10 | 3.6 | 4.52E-02 |
|  | APC/C-mediated degradation of cell cycle proteins | 13 | 3.4 | 2.48E-02 |
|  | Regulation of mitotic cell cycle | 13 | 3.4 | 2.79E-02 |
|  | Metabolism of polyamines | 13 | 3.52 | 2.36E-02 |
|  | Metabolism of amino acids and derivatives | 32 | 2.75 | 4.67E-04 |
|  | Metabolism | 156 | 1.97 | 5.83E-12 |
|  | APC/C:Cdh1 mediated degradation of Cdc20 and other APC/C:Cdh1 targeted proteins in late mitosis/early G1 | 11 | 3.47 | 4.62E-02 |
|  | Cdc20:Phospho-APC/C mediated degradation of Cyclin A | 11 | 3.42 | 4.54E-02 |
|  | APC:Cdc20 mediated degradation of cell cycle proteins prior to satisfation of the cell cycle checkpoint | 11 | 3.38 | 4.83E-02 |
|  | APC/C:Cdc20 mediated degradation of mitotic proteins | 11 | 3.33 | 4.66E-02 |
|  | Activation of APC/C and APC/C:Cdc20 mediated degradation of mitotic proteins | 12 | 3.59 | 2.58E-02 |
|  | MAPK6/MAPK4 signaling | 13 | 3.4 | 2.63E-02 |
|  | Signal Transduction | 138 | 1.45 | 5.31E-03 |
|  | ABC-family proteins mediated transport | 15 | 3.35 | 1.39E-02 |
|  | Transport of small molecules | 63 | 2.06 | 1.63E-04 |
|  | Metabolism of water-soluble vitamins and cofactors | 16 | 3.26 | 1.33E-02 |
|  | Metabolism of vitamins and cofactors | 21 | 3.14 | 4.21E-03 |
|  | Switching of origins to a post-replicative state | 13 | 3.25 | 3.12E-02 |
|  | Synthesis of DNA | 15 | 2.88 | 3.48E-02 |
|  | DNA Replication | 15 | 2.7 | 4.87E-02 |
|  | PTEN Regulation | 14 | 2.8 | 4.50E-02 |
|  | Cellular responses to stress | 29 | 2.29 | 1.31E-02 |
|  | Cellular responses to external stimuli | 31 | 1.98 | 4.47E-02 |
|  | Antigen processing: Ubiquitination & Proteasome degradation | 31 | 2.26 | 1.14E-02 |
|  | Class I MHC mediated antigen processing & presentation | 32 | 2.11 | 2.33E-02 |
|  | Gene expression (Transcription) | 56 | 1.61 | 4.93E-02 |
|  | Unclassified | 547 | 0.75 | 4.43E-26 |
