# Supplementary material for: Genome-Wide Characterization of Alternative Splicing Events and Their Responses to Cold Stress in Tilapia
Source: Front Genet. 2020 Mar 18;11:244. doi: 10.3389/fgene.2020.00244 (PMC7093569; doi:10.3389/fgene.2020.00244)
Supplement: Supplementary file 8 [file Table_8.DOC]

**Additional file 8.** **Overrepresentation of DEGs in categories of PANTHER protein class**

| **Tissue** | **PANTHER Protein Class** | **Gene Number** | **Fold Enrichment** | **FDR** |
| --- | --- | --- | --- | --- |
| Brain | transaminase | 8 | 4.5 | 2.64E-02 |
|  | mRNA splicing factor | 22 | 2.56 | 7.53E-03 |
|  | mRNA processing factor | 27 | 2.27 | 1.04E-02 |
|  | RNA binding protein | 87 | 1.76 | 1.79E-04 |
|  | isomerase | 16 | 2.43 | 4.90E-02 |
|  | dehydrogenase | 34 | 2.13 | 4.75E-03 |
|  | oxidoreductase | 72 | 1.92 | 1.25E-04 |
|  | Unclassified | 1036 | 0.91 | 8.12E-04 |
|  | transcription cofactor | 21 | 0.52 | 3.42E-02 |
|  | C2H2 zinc finger transcription factor | 17 | 0.38 | 4.11E-04 |
|  | serine/threonine protein kinase receptor | 5 | 0.17 | 4.23E-05 |
|  | protein kinase | 28 | 0.54 | 1.44E-02 |
| Heart | transaminase | 7 | 7.01 | 8.00E-03 |
|  | metalloprotease | 21 | 2.37 | 1.73E-02 |
|  | oxidoreductase | 40 | 1.9 | 8.49E-03 |
|  | RNA binding protein | 50 | 1.81 | 7.57E-03 |
|  | Unclassified | 567 | 0.89 | 4.02E-03 |
|  | serine protease | 10 | 0.39 | 3.10E-02 |
|  | C2H2 zinc finger transcription factor | 7 | 0.28 | 3.81E-03 |
|  | serine/threonine protein kinase receptor | 2 | 0.12 | 5.21E-03 |
